# Clinical and quality of life consequences of regimen switching delays in HIV management: a stratified cohort analysis

**DOI:** 10.1038/s41598-025-28209-w

**Published:** 2025-11-29

**Authors:** Basavaraj Poojar, Anusha Natraju, Vijayalakshmi, Satish Rao

**Affiliations:** 1 Department of Pharmacology, Faculty of Medicine, Manipal University College Malaysia (MUCM), Bukit Baru, Melaka, 75150 Malaysia; 2 Department of General Medicine, Kasturba Medical College Mangalore, Manipal Academy of Higher Education, Manipal, India

**Keywords:** HIV/AIDS, ART, EQ-5D-5L, PLWHA, Immunology, Microbiology, Diseases, Health care, Medical research

## Abstract

Health-related quality of life (HRQoL) in people living with HIV/AIDS (PLWHA) is shaped by a combination of HIV-related and non-HIV-related factors, including immunological and virological failure, HIV-associated comorbidities, and antiretroviral therapy (ART)-induced toxicity. Although HRQoL tends to improve during the first year of combination ART (cART), especially among those with advanced HIV, disparities persist. This cross-sectional analytical study assessed HRQoL in 321 PLWHA, of whom 310 were included in the final analysis. HRQoL was evaluated using the EQ-5D-5 L index. Of the participants, 121 (37.7%) had a high EQ-5D-5 L index value of 1, whereas 181 (58.9%) had an index value of less than 1. A statistically significant positive correlation was found between the EQ-5D-5 L index and the recent CD4-T cell count (rs = 0.162, *p* = .004). Gender-based analysis showed males (0.85 ± 0.15) had significantly higher HRQoL scores compared to females (0.77 ± 0.16). Furthermore, delayed switching to second-line regimens was associated with suboptimal recovery in HRQoL. These findings highlight the importance of timely clinical decisions in switching the ART to a more potent regimen especially in resource poor settings.

## Introduction

Globally, at the end of 2023, 42.3 million people were living with Human Immunodeficiency Virus (HIV) infection. Of this 77% (61–89%) million were able to access antiretroviral therapy^1^. With the introduction of combination antiretroviral treatment (cART) the quality of life of people living with HIV and acquired immunodeficiency syndrome (PLWHA) has improved significantly^2–4^.

The World Health Organisation (WHO) defines health as “A state of complete physical, mental and social wellbeing and not merely the absence of disease or infirmity”^5^. Quality of life in a person living with HIV depends on several factors. It could be HIV related or non-HIV related. HIV related factors like immunological and virological failure, HIV associated infective and non-infective comorbidities and toxicity to anti-retroviral drugs are particularly influential^6,7^. Among non-HIV related factors, lack of social support, stigma, poor economic state, partners violence, substance abuse, homelessness, and difficulty in ability to manage the stress and anxiety associated with HIV are important. Due to these factors, the quality of life of PLWHA is lower compared to the general population^8,9^. Studies have also indicated at gender disparity in HRQoL^10^.

Health related quality of life (HRQoL) is a term that refers to an individual’s understanding of how illness or intervention impacts the physical, psychological, and social facets of a person’s health. HRQoL varies over time in a person infected with HIV. It improves in most of the PLWHA during the first year of cART, but the change is more dramatic in those with advanced HIV^4^. A poor HRQoL due to social, neuropsychiatric, economic and substance abuse may have a negative impact on the adherence to HIV treatment and its outcome^11^.

An ever-increasing number of PLWHA on cART has resulted in a large number of them developing treatment failure requiring a switch to a more potent regimen. There are many international and national guidelines to help HIV care providers to make a switch in the treatment regimen, based on treatment failure criteria^12^. However, in resource-limited settingssuch as the public sector hospitals and ART clinics funded by government where this study was conducted,there are often challenges including the unavailability of CD4 T cell count or viral load testing, non-availability or non-affordability of second-line drugs, delayed referral and several patient-related barriers. As a result of this delayed switch in cART, patients may continue for significantly long periods on a failing first line regimen. During this period, the HRQoL declines sharply due to increased morbidity associated with poor immunity. This has immediate and delayed implications, including an adverse impact on the efficacy of the new cART regimen after the delayed switch. This study aims to evaluate and characterize the health-related quality of life (HRQoL) among people living with PLWHAon cART using the EQ-5D-5 L questionnaire and EQ-VAS score. Specifically, the study seeks to compare demographic variables, social factors, and other HIV-related characteristics that may influence HRQoL across three distinct cART groups(stable on first line, first line treatment failure and stable second line treatment). By exploring these factors, the research intends to provide insights into the impact of delayed regimen switching on immune reconstitution and its potential implications for HRQoL outcomes.

## Methodology

This cross-sectional analytical study was conducted in the outpatient and inpatient departments of hospitals affiliated with Kasturba Medical College, Mangalore.

### Sample size calculation

The sample size was calculated using a formula for frequency estimation in a finite population, as described below. The calculation was performed using the OpenEpi version 3 open-source calculator.

$$\:n=\frac{DEFF\times\:N\times\:p\left(1-p\right)}{\begin{array}{c}{d}^{2}+{Z}_{1}^{2}-\alpha\:/{2}^{\times\:}\left(N-1\right)\times\:p\left(1-p\right)\\\:\frac{\:\:\:\:\:\:\:\:\:\:\:\:\:\:\:\:\:\:\:\:\:\:\:\:\:\:\:\:\:\:\:\:\:\:\:\:\:\:\:\:\:\:\:\:\:\:\:}{N}\end{array}}$$

Where:

- **DEFF**: Design effect (assumed to be 1 for this study, as the sampling technique was simple non-random).
- **N**: Population size (1000, based on the registry data).
- **p**: Estimated proportion of the outcome (low HRQoL) in the population (50% ± 5%).
- **d**: Desired margin of error (5%).
- **Z_{1-\alpha/2}**: Z-score for the desired confidence level (1.96 for a 95% confidence level).
- **N-1**: Finite population correction factor.

Based on these assumptions, the required sample size was calculated to be 310 participants. To account for a potential non-response rate of 10%, the final target sample size was adjusted accordingly.

**Sampling technique: **A simple non-random sampling technique was employed for participant selection. This method involved selecting PLWHA on cART who had complete data available for the study, without repetition.

The study was conducted over, a period of two years from August 2018 to July 2020.

### Patient selection criteria

Participants eligible for inclusion in the study were individuals who were HIV positive and on combination antiretroviral therapy (cART) for a minimum duration of one year. Participants were required to be between the ages of 18 and 60 years, and they needed to be attending regular follow-up visits at the participating hospitals. In addition, participants had to be willing to provide informed consent for participation in the study.

Exclusion criteria included: active opportunistic infections at the time of enrollment or a history of major illness, drug-related adverse events, or opportunistic infections within the six months prior to the HRQoL assessment. Participants with documented poor adherence to cART were also excluded, Individuals with malignancies or terminal illnesses with an expected survival of less than six months were excluded. Similarly, participants with advanced HIV disease or HIV-related comorbidities, whose expected survival was less than six months, were not included. Individuals on failing second-line cART or those receiving third-line cART were excluded, Lastly, pregnant women were excluded from the study to avoid any potential confounding effects related to pregnancy on HRQoL. We have provided the operational definitions used in the study as Supplementary Material 1.

### Data collection methodology

The study was approved by the Institutional Ethics Committee of Kasturba Medical College, Mangalore, India, and necessary permissions were obtained from the respective hospital superintendents. All research procedures were conducted in accordance with the ethical standards of the Declaration of Helsinki. Informed consent was obtained from all participants and/or their legally authorized representatives prior to enrolment. Permission was also obtained from EuroQol research foundation (based at Marten Meesweg, 107 3068 AV Rotterdam, The Netherlands) to use the patented EQ-5D-5 L questionnaire and EQ-VAS for the purpose of this study. Study related procedures and enrolment of eligible subjects was done after obtaining an informed consent. Confidentiality of patient’s details was maintained at all levels using appropriate coding.

Subjects fulfilling inclusion and exclusion criteria were enrolled during their routine follow up visits to the hospital. Based on the data collected from hospital records PLWHA were screened for enrolment into the study. After the enrolment, every patient was interviewed to obtain medical history and were subjected to a one-time physical examination. Data related to demography, history, physical examination, investigations, socioeconomic status was classified using the modified Kuppuswamy scale^13^ and EQ-5D-5 L questionnaire with EQ-VAS score details were collected using a semi structured proforma. In addition to variables directly related to HRQoL, data on comorbidities such as hypertension, diabetes, and smoking status were collected to provide a comprehensive clinical profile of the study population, given the increasing relevance of multimorbidity in ART-treated individuals.

All enrolled subjects were administered the EQ-5D-5 L questionnaire in a language preferred by them. The health-related quality of life was measured based on EQ-5D-5 L index value. The EQ-VAS score, representing the current HRQoL as perceived by the patient was captured separately.

**Tools used in the study**:

A. Edmonton’s frail score: It has nine domains (cognition, general health status, functional independence, social support, Medication use, Nutrition, Mood, continence, Functional performance)^14^. It has a maximum score of 17 and a minimum score of zero. Scoring was done by adding the points for each domain. A score of 0–5 was considered not frail, 6–7 as vulnerable, 8–9 as mild frailty,10–11 moderate frailty and 12–17 as severe frailty. For analysis purpose a score of 0–5 was considered as not frail and a score of > 5 was considered as frail.
B. Scoring of EQ-5D-5 L Questionnaire and EQ-VAS^16,15^.

The EQ-5D-5 L questionnaire was employed to assess the health-related quality of life (HRQoL) of participants, using its five-dimensional descriptive system—mobility, self-care, usual activities, pain/discomfort, and anxiety/depression. Each domain was rated on a five-point Likert scale, and responses were combined into a 5-digit health state code (e.g., 11111). Incomplete or ambiguous responses were coded as ‘9’.

In addition to this, the EQ Visual Analogue Scale (EQ-VAS) was used to capture participants’ self-perceived overall health status on a 0–100 scale, with 0 representing the worst imaginable health and 100 the best. This tool complemented the EQ-5D-5 L to provide a holistic picture of patient well-being.

For scoring, a summary index was generated using the crosswalk link function developed by Van Hout et al. (2012), following the EuroQol Research Foundation guidelines. This index provides a value between 0 and 1, with 1 indicating full health and values less than 1 reflecting varying levels of impairment.

A more detailed description of the scoring methodology and sample calculation examples have been provided in Supplementary Material 1 for reference.

### Statistical analysis

The descriptive statistics are presented as mean (± std) for normally distributed continuous variables and as median (IQR) for variables with wide standard deviation. Categorical variables are presented as proportion (percentage).

Calculated EQ-5D-5 L index value and EQ-VAS score were compared between the groups for statistical significance. Other independent variables related to demography, social, history, physical examination, were also compared between the three cART groups.

Observed difference between the groups were analysed for statistical significance using one-way ANOVA or Kruskal Wallis H test for continuous variables and Chi square test for categorical variables. Tukey post hoc tests (multiple comparisons) were done to detect the difference in variables between the individual cART groups. For Kruskal Wallis H test, post hoc analysis were performed using Dunn’s (1964) procedure. A Bonferroni correction for multiple comparisons was made using the adjusted “p” value.

The CD4-T cell count changes over a period of time, (CD4-T cell count at HIV diagnosis, CD4-T cell count at 18,12,6 months prior to enrolment) were recorded and analysed appropriately. Two-way ANOVA was conducted to examine the effect of interaction between the gender and cART groups on EQ-5D-5 L index value/EQ-VAS score.

Spearman’s correlation analysis was done to assess correlation between continuous independent variables, likely to influence HRQoL and EQ-5D-5L index value/ EQ-VAS score. For regression analysis study subjects were divided into those with high EQ-5D-5L (1) index value and those with low EQ-5D-5L index value (< 1).Variables found to be statistically significant by univariate or bivariate analysis, were subjected to a binary logistic regression analysis to determine independent predictors of low EQ-5D-5L index value. A” p” value of < 0.05 was considered statistically significant. Data was analysed using SPSS-27 software.

## Results

At the end of the recruitment period, there were about 780 patients on active treatment. Based on the inclusion and exclusion criteria, 321 patients were enrolled into the study. Patients who were lost to follow-up were excluded from analysis. Out of the 321 patients, 11 patients (3.4%) could not be administered the quality of life and frailty instruments, so had no HRQoL data. At the end of the study, of the 310 patients with complete data, 121 (37.7%) had a high EQ-5D-5 L index value of 1, and 181 (58.9%) had an low EQ-5D-5 L index value of less than 1.The missing value proportion was < 2% for quantitative variables. There were no missing data for categorical variables related to demography and co-morbidities. The missing quantitative data were replaced with series mean. The demographic characteristics of the participants are summarized in Table 1a. Treatment history and immunovirological profiles are presented in Supplementary Table 1b. Health-related quality of life outcomes are shown in Table 2.

**Table 1:** Demography details.

Variable	Total	Variable	Total
**Age**: Mean age in years **Age groups** < 30yrs 31-40yrs 41-50yrs 51-60yrs	43.55(± 8.8) 29(9%) 82(25.5%) 149(46.4%) 61(19.1%)	**Smoking:** Yes No	18(5.6%) 303(94.4%)
**Gender** Female Male	137(42.7%) 184(57.3%)	**Hypertension:** Yes No	31(9.7%) 290(90.3%)
**Residence** Rural Urban	146(45.5%) 175(54.5%)	Diabetes: Yes No	13(4%) 308(96%)
**Education:** Illiterate + Primary Secondary + Graduate	118(36.8%) 203(63.2%)	**Chronic kidney disease:** Yes No	7(2.2%) 314(97.8%)
**Economic status:** Upper class Upper middle class Lower middle class Upper lower class	73(22.7%) 105(32.7%) 117(36.4%) 26(8.1%)	**Number of comorbidities(CM):** No CM One CM > One CM	268(83.5%) 43(13.4%) 10(3.1%)
**Marital status:** Married Unmarried	268(83.5%) 53(16.5%)	**Frailty:** Yes No	17(5.3%) 304(94.7%)
BMI in Kg/m^2^:	22.10(± 10.22)		

**Table 2:** Health related quality of life.

Variable	Total *n* = 310(100%) *n* (%) Missing (11)	cART groups			X^2^ & *p* Value
		First line stable *n* = 197 Missing (6)	First line failure & early second line *n* = 29 Missing (2)	Second line stable *n* = 84 Missing (3)	
**EQ-5D-5 L Domains:**					
**1. Mobility**:					
No problem	293(94.5%)	186(94.4%)	26(89.7%)	81(96.4%)	LR X^2^ = 1.74 *p* = .419
Any problem	17(5.5%)	11(5.6%)	3(10.3%)	3(3.6%)	
**2.Self-care:**					
No problem	296(95.5%)	189(95.9%)	27(93.1%)	80(95.2%)	LR X^2^ = 0.44 *p* = .803
Any problem	14(4.5%)	8(4.1%)	2(6.9%)	4(4.8%)	
**3.Usual activity:**					
No problem	220(71%)	142(72.1%)	18(62.1%)	60(71.4%)	X^2^ = 1.24 *p* = .537
Any problem	90(29%)	55(27.9%)	11(37.9%)	24(28.6%)	
**4.Pain/discomfort**:					
No problem	157(50.6%)	114(57.9%)	6(20.7%)	37(44%)	X^2^ = 15.99 *p* = < 0.001*
Any problem	153(49.4%)	83(42.1%)	23(79.3%)	47(56%)	
**5.Anxiety/depression:**					
No problem	181(58.4%)	125(63.5%)	14(48.3%)	42(50%)	X^2^ = 5.73 *p* = .057
Any problem	129(41.6%)	72(36.5%)	15(51.7%)	42(50%)	
**EQ-5D-5 L Index value:**	0.82(± 0.16)	0.84(± 0.16)	0.74(± 0.14)	0.79(± 0.17)	*p* = .004** *p* = .005**
**EQ - VAS:**	74.21(± 9.74)	75.57(± 9.02)	71.95(± 10.62)	71.80(± 10.52)	

*Significant by Chi-square test.

**Significant by one-way ANOVA.

### Correlation between HRQoL metrics and CD4-T cell count

**Fig. 1:**
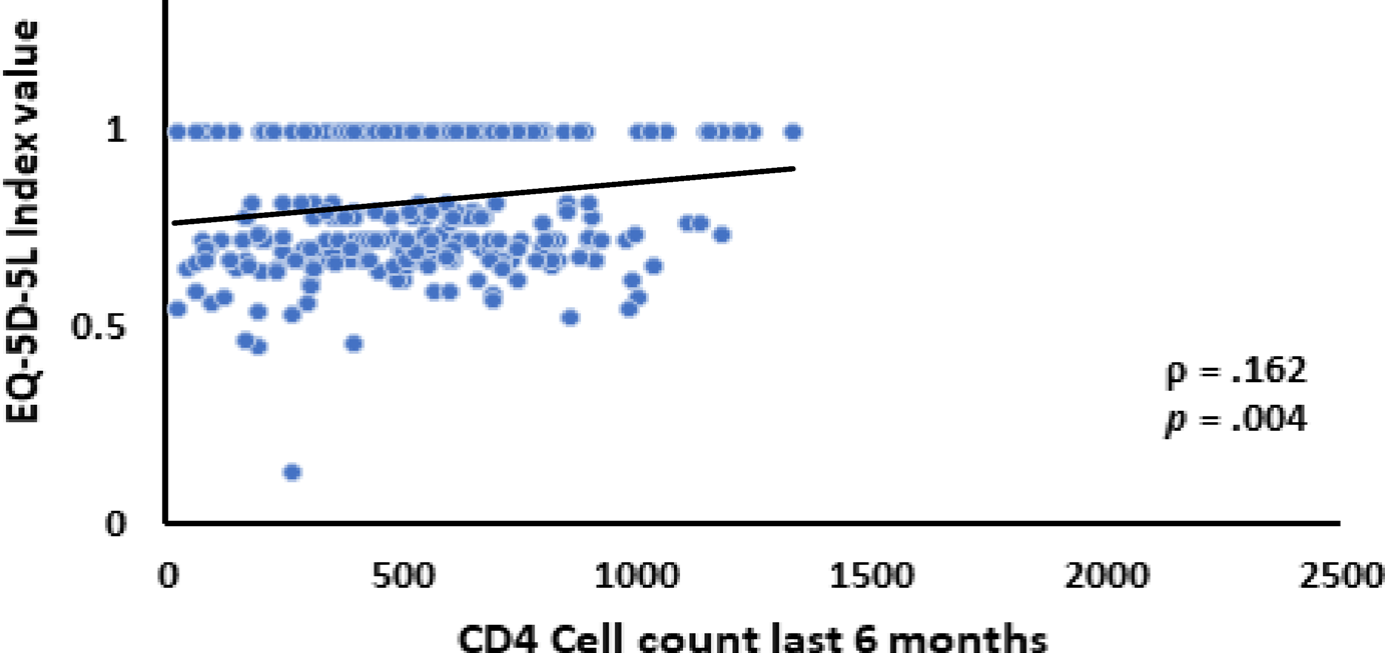
Correlation Between EQ-5D-5 L Index Value and CD4-T Cell Count.

**Fig. 2:**
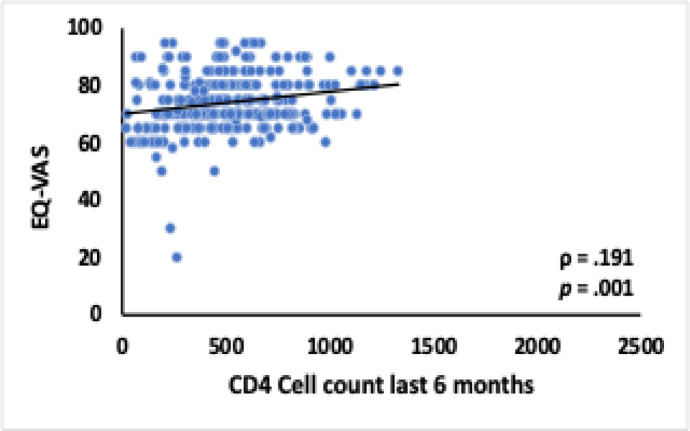
Correlation Between EQ-VAS Score and CD4-T Cell Count.

A statistically significant positive correlation was observed between the EQ-5D-5 L index value and the CD4-T cell count measured within the past six months (rs = 0.162, *p* = .004), as depicted in Fig. 1. Additionally, the EQ-VAS score exhibited a significant positive correlation with the CD4-T cell count during the same period (rs = 0.191, *p* = .0.01), as illustrated in Figs. 1 and 2. These findings highlight the relationship between improved health-related quality of life (HRQoL) metrics and higher immune cell counts among participants.

### Correlation of Health-Related quality of life metrics with baseline clinical parameters

**Fig. 3:**
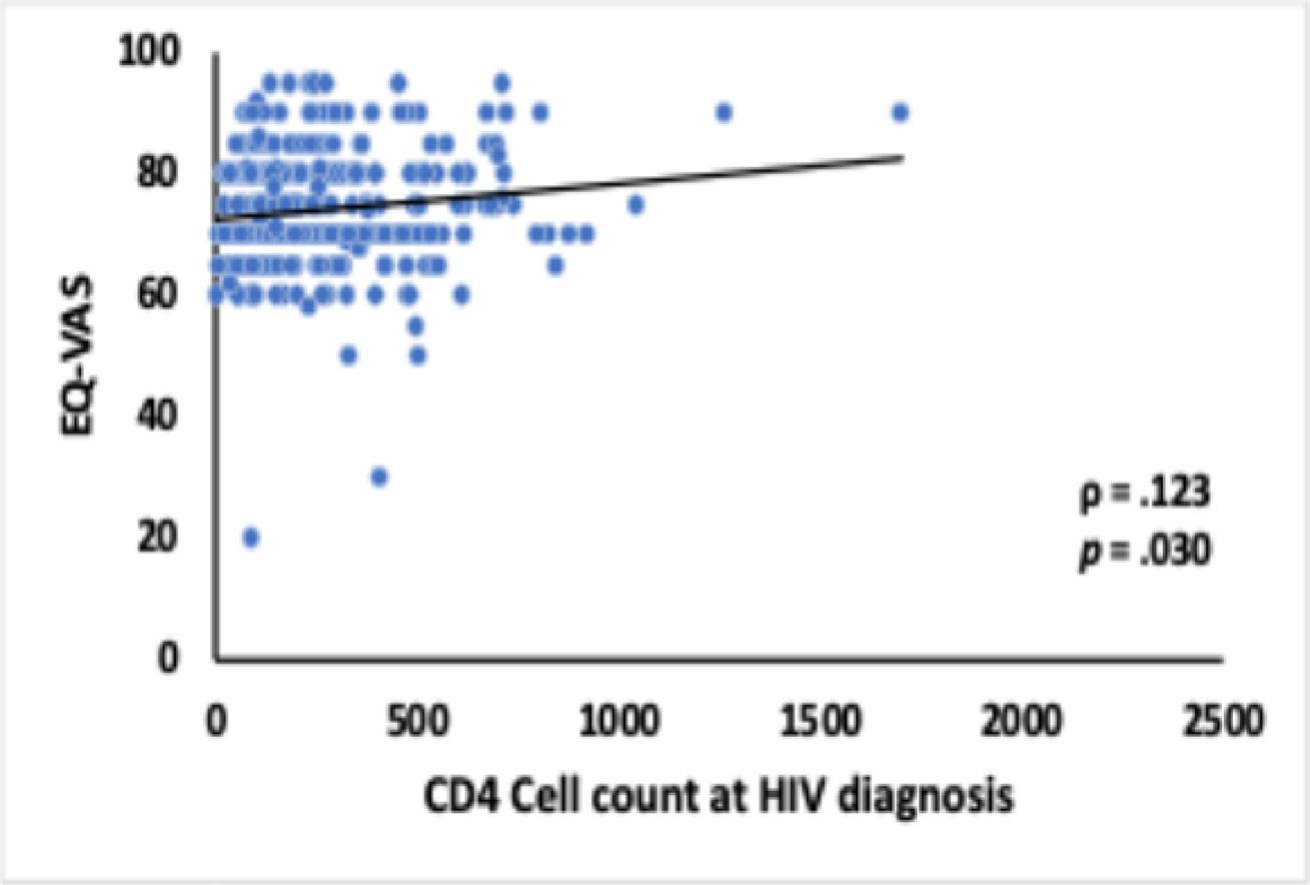
Correlation Between EQ-VAS Score and CD4-T Cell Count at HIV Diagnosis.

**Fig. 4:**
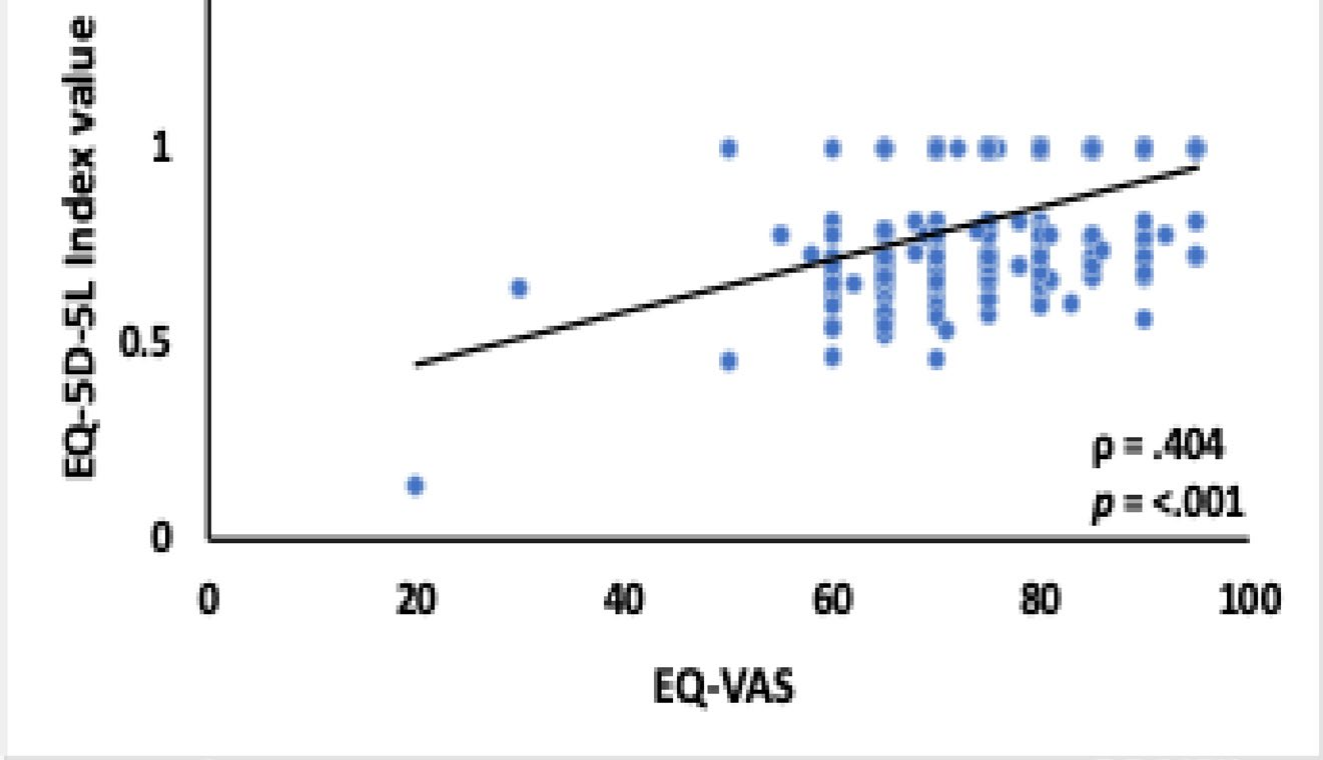
Correlation Between EQ-5D-5 L Index Value and EQ-VAS Score.

A statistically significant positive correlation was observed between the EQ-VAS score and the CD4-T cell count at the time of HIV diagnosis, r_s_(308) = 0.123,*p* = .0.30 Additionally, there was a strong statistically significant positive correlation between the EQ-5D-5 L index value and the EQ-VAS score, r_s_(308) = 0.404,*p* < .0.001. These findings are illustrated in Figs. 3 and 4, respectively, highlighting the association between baseline clinical parameters and health-related quality of life metrics in PLWHA.

**EQ-5D-5 L index value vs age groups: **A one-way ANOVA was performed to examine the significance of the differences in EQ-5D-5 L index values across the various age groups. The results indicated that the observed differences in index values between the age groups were statistically nonsignificant.

**Fig. 5:**
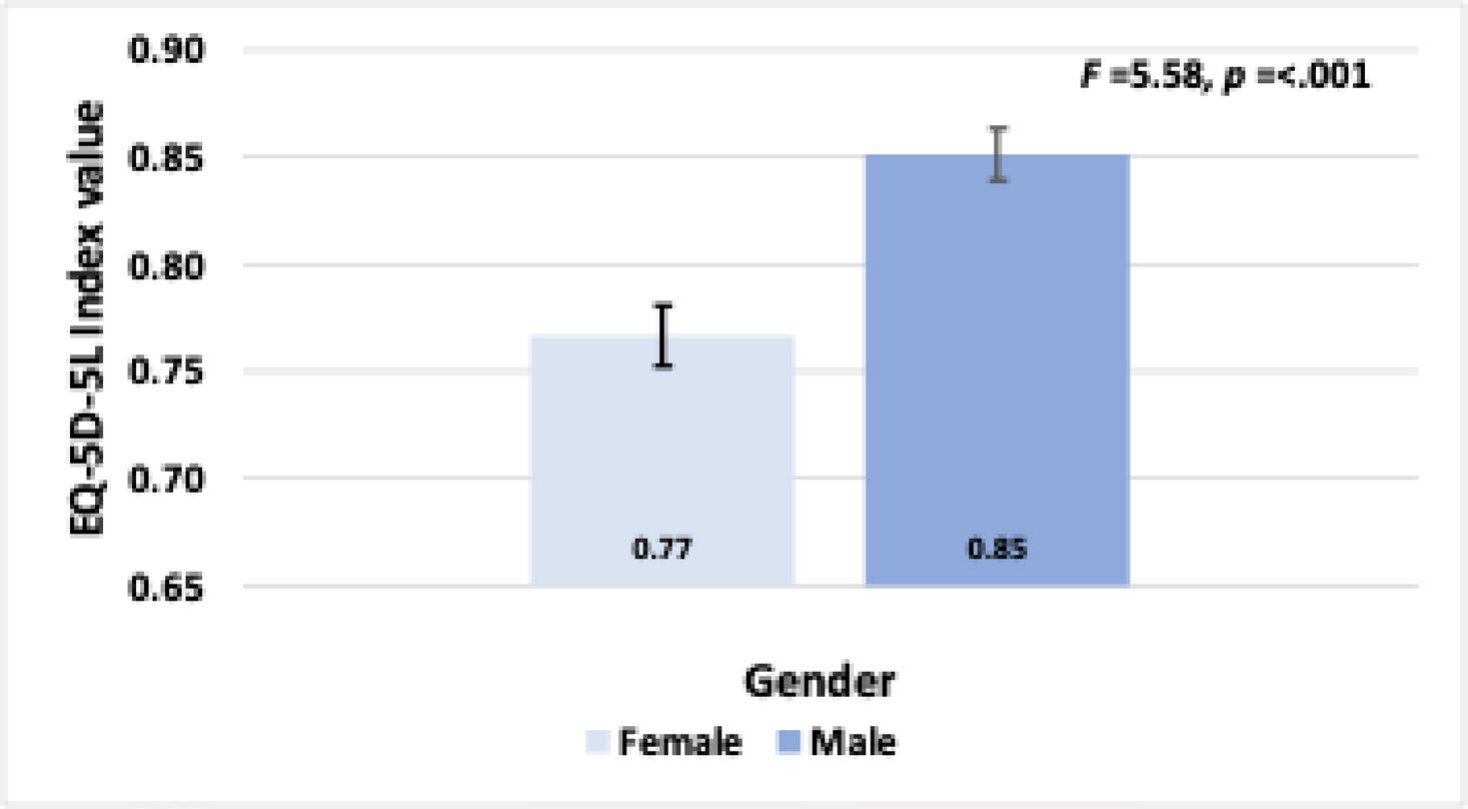
illustrates the comparison of EQ-5D-5 L index values between males and females.

**EQ-5D-5 L index value vs gender: **An independent-samples t-test was conducted to assess whether there were differences in EQ-5D-5 L index values between males and females. The results revealed a statistically significant difference, with males (0.85 ± 0.15) scoring higher than females (0.77 ± 0.16). The mean difference was − 0.085 ± 0.02, with an F-value of 5.58 and a p-value of < 0.001.( see Fig. 5 illustrates the comparison of EQ-5D-5 L index values between males and females).

Chi-square tests of independence were performed to assess associations between the levels of each EQ-5D-5 L domain and cART groups.

**Fig. 6:**
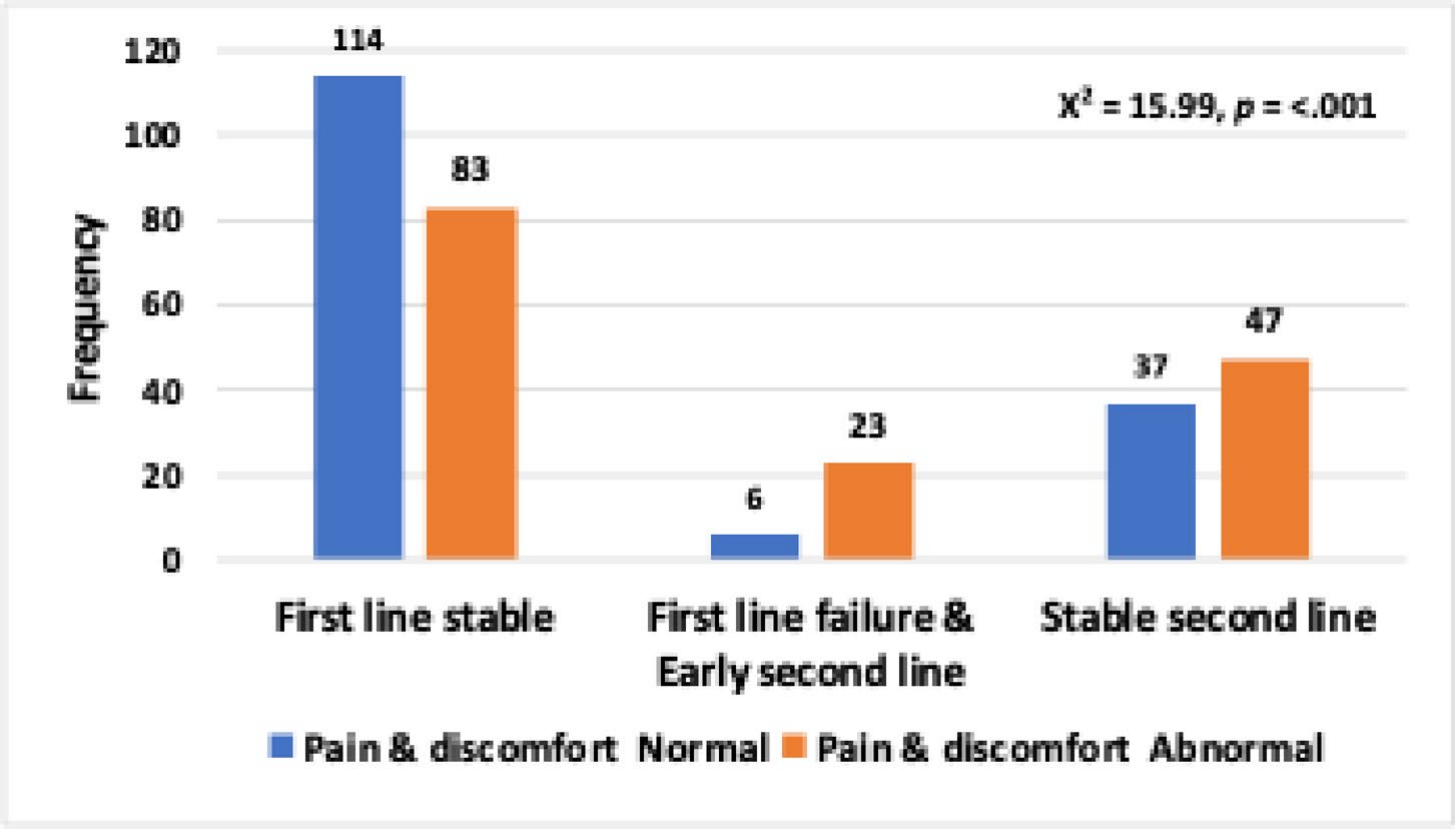
Frequency of pain and discomfort levels across different treatment stages and according to abnormality status. The bar chart illustrates the distribution of patients experiencing pain and discomfort (blue bars) versus those with normal levels (orange bars) within three treatment categories: “First line stable,” “First line failure & Early second line,” and “Stable second line.” The chi-square test statistic (χ2 = 15.99) and associated p-value (*p* < .001) indicate a statistically significant association between treatment stage/abnormality status and the presence of pain and discomfort.

- **Mobility**: The association between mobility levels and cART groups Mobility level 1 (normal mobility) was compared with the abnormal mobility levels (2–5).The likelihood ratio (LR) chi-square test revealed no significant relationship (LR/χ² = 1.74, *p* = .419).
- **Self-care**: Similarly, no significant association was found between the self-care domain levels and cART groups (LR/χ^2^ = 0.44, *p* = 0.803).
- **Usual activity**: The analysis for usual activity levels showed no significant association with cART groups. Level 1 (normal usual activity) was compared to abnormal levels (2–5), with the chi-square test yielding a nonsignificant result (LR/χ^2^ = 1.24, *p* = 0.537).
- **Pain and discomfort**: A significant association was detected between pain and discomfort levels and cART groups. Level 1 (no pain/discomfort) was compared with levels 2–5 (increasing pain/discomfort). The chi-square test showed a significant result (χ^2^ = 15.99, *p* < .001). As shown in Fig. 6, there was a significant variation in pain and discomfort levels across different treatment stages and abnormality status, with higher levels reported among patients in the “First line failure & Early second line” group.

Chi-square test of independence was conducted between the Pain & discomfort domain levels and cART groups. Pain & discomfort level 1 was considered normal. Levels 2,3,4 and 5 were considered abnormal. There was a significant association (X^2^ = 5.73, *p* = .057).

**Fig. 7:**
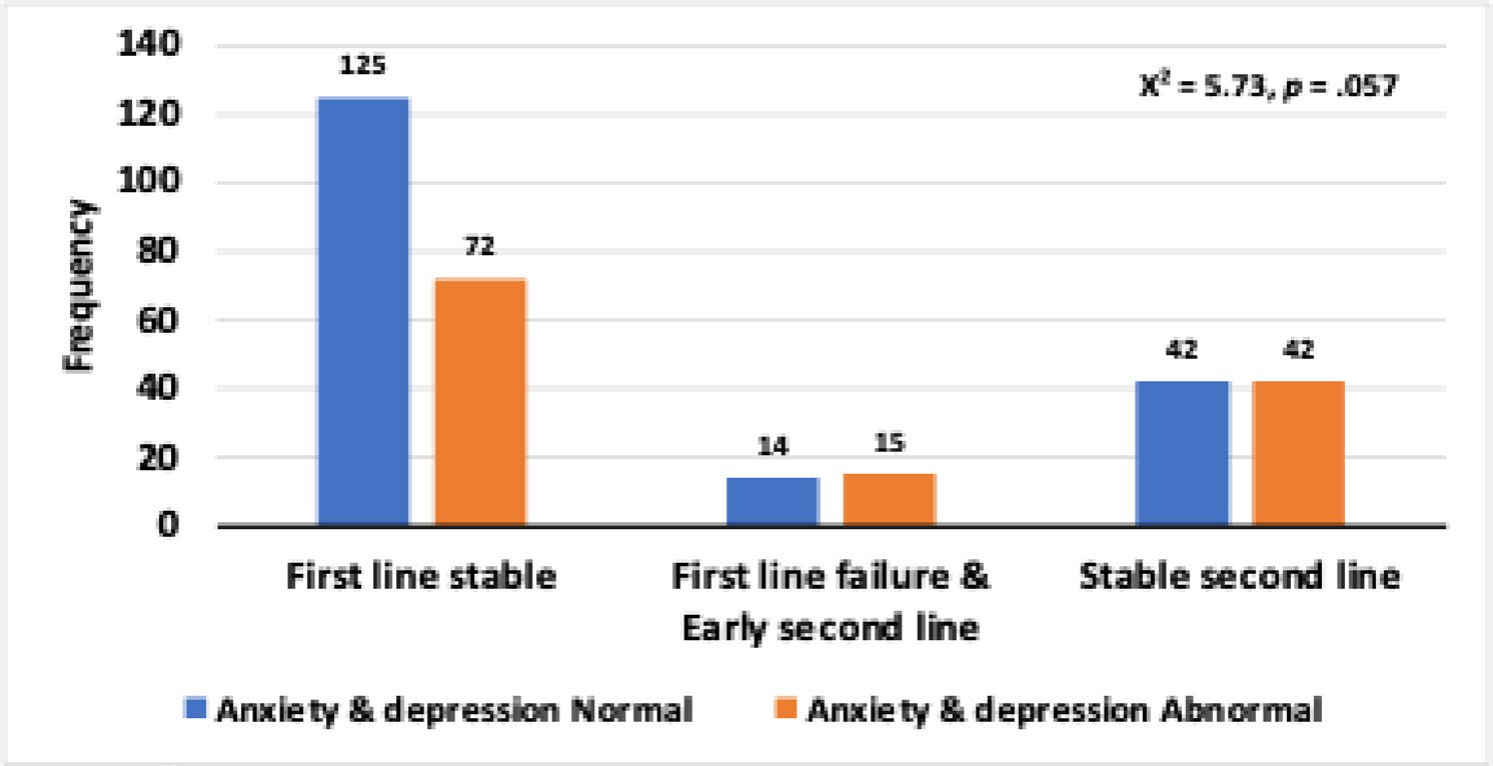
Distribution of anxiety and depression levels in relation to treatment stage and abnormality status. This bar chart presents the frequency of patients with normal (blue bars) and abnormal (orange bars) levels of anxiety and depression across three treatment phases: “First line stable,” “First line failure & Early second line,” and “Stable second line.” The chi-square test results (χ2 = 5.73, *p* = .057) suggest a trend towards an association between these variables, although the p-value is slightly above the conventional significance threshold of 0.05.

**Anxiety and depression: **A marginally significant association was found between anxiety/depression levels and cART groups. Level 1 (no anxiety/depression) was compared with levels 2–5 (increasing anxiety/depression). The test approached significance (χ^2^ = 5.73, *p* = 0.057). As shown in Fig. 7, the distribution of anxiety and depression levels varied across treatment stages, with a trend toward statistical significance (*p* = .057) observed between abnormality status and treatment phase.

### EQ-VAS scores and their associations with demographic and cART groups

The mean EQ-VAS score was 74.21 ± 9.74, with a median score of 75 (IQR 70–80). The minimum and maximum EQ-VAS values recorded were 20 and 95, respectively. Notably, 121 participants (39.03%) had EQ-VAS scores in the range of 60–70.

**EQ-VAS vs. gender: **An independent-samples t-test was conducted to examine potential differences in EQ-VAS scores between males and females. The results indicated no statistically significant difference in the scores between genders. Males had a mean score of 74.92 ± 9.36, while females scored 73.25 ± 10.18. The mean difference between males and females was − 1.67 ± 1.11, with an F-value of 0.21 and a p-value of 0.136.

**EQ-VAS vs. age groups: **A one-way ANOVA was performed to assess the significance of differences in EQ-VAS scores across various age groups. The results showed no statistically significant difference suggesting that age did not have a significant effect on the self-reported health status as measured by the EQ-VAS.

**Fig. 8:**
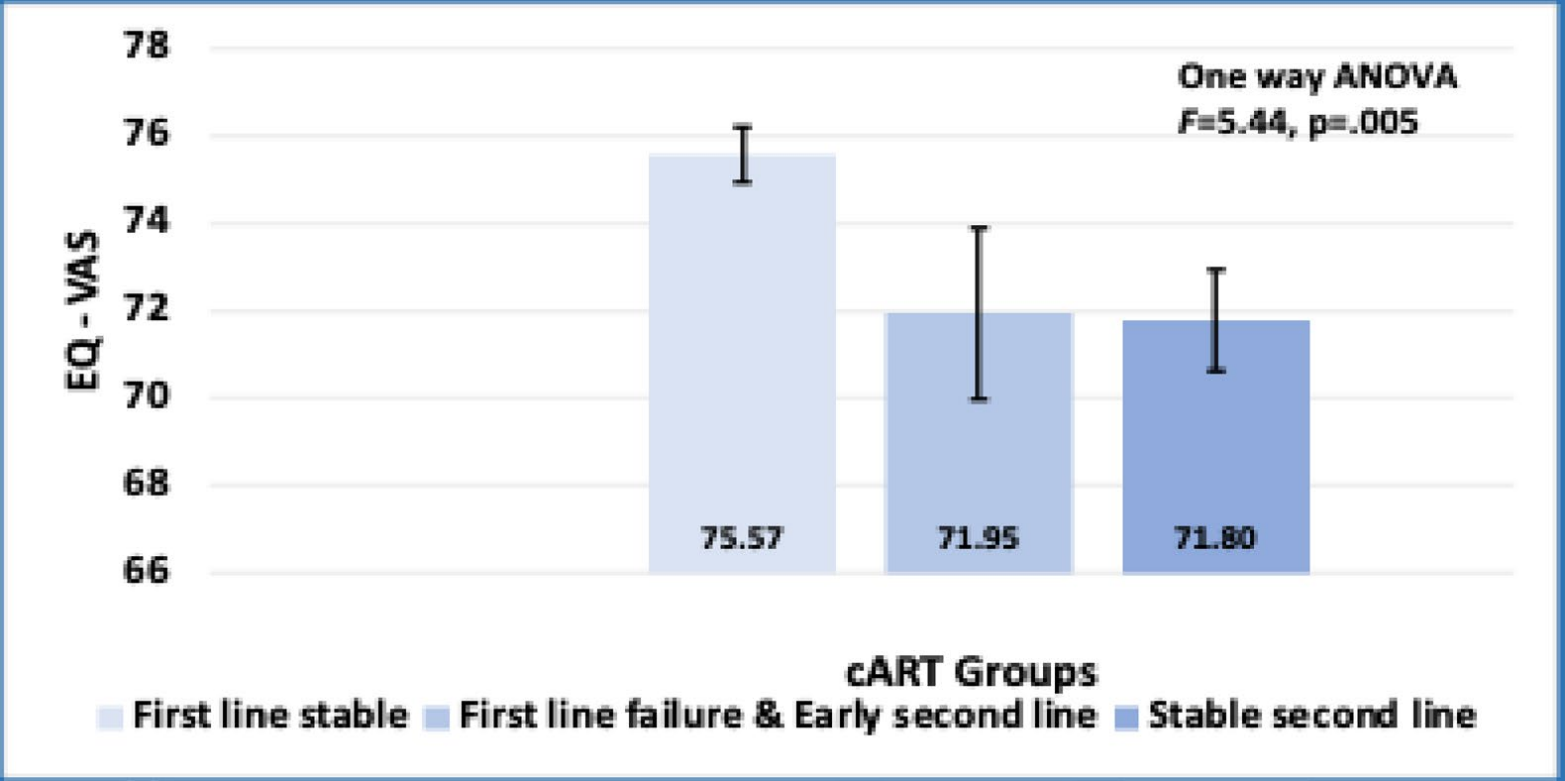
Mean EQ-VAS scores across different cART groups. The bar graph displays the mean scores on the EQ-VAS (EuroQol visual analogue scale) for three cART groups: “First line stable,” “First line failure & Early second line,” and “Stable second line.” Error bars represent the standard error of the mean. Results from a one-way ANOVA indicate a statistically significant difference in mean EQ-VAS scores between the groups (F = 5.44, *p* = 0.005).

**EQ-VAS vs. cART groups: **A one-way ANOVA was also conducted to determine whether there were significant differences in EQ-VAS scores between different cART treatment groups. The analysis revealed a statistically significant difference in EQ-VAS scores among the cART groups (F = 5.44, *p* = .005), indicating that cART treatment groups were associated with differing self-reported health outcomes. As illustrated in Fig. 8, the mean EQ-VAS scores differed significantly among the cART groups, with the “First line stable” group reporting higher perceived health status compared to others.

### Binary logistic regression analysis of predictors of low HRQoL

A binary logistic regression was performed to assess the effects of age, gender, residence, and the CD4-T cell count from the last 6 months on the likelihood of low HRQoL (defined as an EQ-5D-5 L index value < 1) among enrolled PLWHA. The logistic regression model was statistically significant, χ^2^(4) = 34.431, *p* < .0.01.

Of the four predictor variables, only gnder and CD4-T cell count in the last 6 months were statistically significant. Females were found to have 2.19 times higher odds of having low HRQoL compared to males (OR = 2.19). Additionally, an increase in the CD4-T cell count over the last 6 months was associated with a reduced likelihood of having low HRQoL, indicating that higher CD4-T cell counts were protective against impaired HRQoL.

## Discussion

The study had proposed to evaluate the adverse impact of delayed switch to second line cART on HRQoL. Among all the PLWHA enrolled with complete HRQoL data, 58.9% had low EQ-5D-5 L index value less than 1. As expected, both EQ-5D-5 L index values and EQ-VAS scores recorded for the PLWHA on first line stable cART were higher compared to the other groups in the study. Studies in the past have also shown improvement in HRQoL following initiation of cART^3,17^. This positive impact of cART on HRQoL is sustained and maintained while a patient is stable on HIV treatment.

71.7% of enrolled patients were late presenters. Despite presenting late to HIV care with late initiation of treatment, majority of PLWHA showed a good response to cART. 78.5% of the PLWHA were on suppressive cART prior to 6 months of their enrolment. At the time of enrolment that proportion increased to 87.5%. Among those on stable first-line cART, 91.1% had achieved a viral load below 40 copies/ml in the 6 months prior to their enrolment, and this proportion further increased to 97.5% at the time of enrolment. Even among the stable second line cART group 92% had the recent viral load below 40 copies/ml. This viral suppressive rate on cART is as per WHO 2020 goal of 90%^18^.

In high-income countries, regular viral load measurement is the standard of care to detect treatment failure and guide decisions related to cART switch. In resource limited settings viral load monitoring may not always be available. A study in resource poor settings of Africa, South America and Asia found that switching to second line cART tends to occur earlier at a higher CD4-T cell counts in HIV care programmes with viral load monitoring^19^. Treatment decisions based on periodic HIV viral load checks as recommended by WHO and other organisations should be strictly followed to necessitate an early switch in regimen, if needed^12^. This prevents further immunological deterioration and decline in HRQoL. This has been confirmed by an earlier study done in Mumbai^20^.

During the study period, there was a notable shift in the backbone NRTIs used in first-line cART. Zidovudine use decreased by approximately 10%, while Tenofovir-based backbones increased correspondingly, likely reflecting updates to the National treatment guidelines^21^. Among the third agents, Atazanavir/Ritonavir remained the preferred choice for second-line regimens, while Efavirenz was more commonly used in earlier regimens.

The observed decrease in zidovudine-based regimens in our study likely mirrors broader national and global transitions in HIV treatment protocols. Over time, evidence has shown that tenofovir offers several advantages over zidovudine, including better virologic control and a reduced likelihood of developing resistance^22^. This shift was guided by evolving WHO recommendations grounded in clinical efficacy, safety data, and field-level implementation feedback^23^. At our study site, previous reports have documented patient intolerance and side effects as key reasons for transitioning away from ZDV-based regimens^24^, a trend echoed by other tertiary centres in India^25^. Moreover, meta-analytic data by Dadi et al. demonstrated that TDF significantly outperformed ZDV in achieving viral load suppression below 50 copies/ml^26^. These trends highlight how updated evidence and policy alignment have directly shaped clinical decision-making at the facility level, ensuring safer, more effective treatment for people living with HIV.

There was a dramatic decline in HRQoL indicators when patients developed treatment failure. Correlation analysis revealed a significant positive correlation between EQ-5D-5 L, and the CD4-T cell count done within the last 6 months. This was further confirmed by logistic regression. CD4-T cell count done within the last 6 months was the only significant HIV related variable which predicted an EQ-5D-5 L index value of < 1. As per the regression coefficient, each unit rise in CD4-T cell count done within the last 6 months decreased the risk of having a low EQ-5D-5 L index value. Earlier studies from India have shown a positive association between CD4-T cell count and HRQoL^27^. This could be easily explained as the CD4-T cell count reflects the immunological status of an individual. A CD4-T cell count below 200 cell/µl is associated with a higher risk of opportunistic infections and other AIDS related non infective conditions. Due to this increased risk of morbidity, there is a decline in HRQoL. Results in this respect agree with other similar published studies^3,28,29^.

Frequency of PLWHA with addictions and comorbidity was lower in the study population compared to data from other similar settings^6^. The relatively low number of participants with substance use issues in our study could be attributed to the inclusion criteria, which favoured individuals with consistent clinic attendance and good adherence to cART. These requirements may have inadvertently excluded patients with active addictions, who often face challenges in maintaining regular follow-up. As a result, this subgroup may be underrepresented in our sample.

The median CD4-T cell count at HIV diagnosis was 219 cells/µl (IQR 119–373). 71.7% of PLWHA had a CD4-T cell count ≤ 350 cells/µl at HIV diagnosis. There was no difference between the three groups in the CD4-T cell count done at HIV diagnosis. The treatment failure group had the lowest median CD4-T cell count only to increase by a small margin in PLWHA on stable second line cART. CD4-T cell change over time was indicative of a pattern in which PLWHA on stable first line cART had a robust increasing trend at all time points but a blunted CD4-T cell count gain in the other two groups. A declining CD4-T cell count trend was observed at least 18 months prior to enrolment in the treatment failure and stable second line cART groups. Early intervention in these two groups could have prevented further immunological deterioration and as a result, a decline in HRQoL. Late diagnosis of treatment failure with a delayed switch has been reported by other researchers^29,30^. This is an emerging issue in the delivery of HIV care^31^. If a patient continues with a failing cART, will transmit the disease with resistant strains. Chances of developing opportunistic infections and other non-infective comorbidities will also increase. There is always a risk of these patients accumulating more resistant strains, which may have an impact on future treatment options.

After switching to an optimal cART regimen, restoration of immunological status and HRQoL may take a longer time to return to pre-existing levels. The EQ-5D-5 L index value increased sharply following a steep decline in the group with treatment failure to first line cART/early second line cART in both males and females. In females, the changes were more marked than in males. Females on second line stable cART had the worst EQ-5D-5 L index values compared to males in the same group. EQ-VAS scores also showed the same trend of decline in both males and females, but in the stable second line cART group, there was a discordant response in EQ-VAS scores between males and females. In females the decline continued from low EQ-VAS scores recorded during first line treatment failure. This indicates a discrepancy between the perceived HRQoL as indicated by the EQ-VAS and the measured HRQoL as indicated by EQ-5D-5 L index value. It could be attributed to the anxiety and uncertainty associated with treatment failure. Most of the females reported being in a stressful state. They had the burden of managing their home and taking care of other family members, despite being sick.

Studies in the past have reported a difference in HRQoL between female and male PLWHA. The social issues related to HIV, economic issues and the cultural background appear to be the dominant factors in females whereas the factors related to HIV treatment and complications, substance abuse appear to be the dominant factors in males^28,32,33^. This study showed a significant difference in EQ-5D-5 L index vales among male and females. Females had consistently lower EQ-5D-5 L index values in all the three groups. The logistic regression indicated at a very significant association. There was no interaction effect of Gender and cART groups on EQ-5D-5 L index value. Despite having a better profile of HIV related indicators, HRQoL in females was low compared to males. This could be due to other non-HIV related factors reported exclusively in females. The social factors tend to affect females more than the males. Present study was not designed to evaluate other HIV related and social factors contributing to this observed difference in EQ-5D-5 L index values.

While both the EQ-5D-5 L index and EQ-VAS scores serve as validated measures of health-related quality of life, the EQ-5D-5 L may better capture domain-specific disparities such as those influenced by gender. In this study, although a strong positive correlation was observed between EQ-VAS and EQ-5D-5 L index values, only the EQ-5D-5 L index revealed statistically significant gender-based differences. This suggests that the EQ-5D-5 L’s structured, multi-domain format may be more sensitive to variations in functional and symptomatic dimensions of HRQoL, whereas the EQ-VAS may reflect broader perceptions of well-being that are less influenced by demographic subgroups.

Among the EQ-5D-5 L domains the difference was seen mainly in the level of pain/discomfort domain between the three groups. There was a trend towards a significant difference in the level of anxiety and depression domain. These findings are consistent with other studies^34^. HIV-related pain/discomfort and treatment complications like adverse drug reactions may be the reason for this observation. Earlier studies have identified factors such as anxiety, depression, stigma, stress of manging the family and partner violence as important social causes contributing to a poor HRQoL^11,33,35^. Early diagnosis and management of underlaying anxiety, stigma and depression improves quality of life and adherence to cART^2,10^. A delayed diagnosis of treatment failure may also be a contributing factor. A person with treatment failure has a higher risk of associated morbidity and mortality. PLWHA in treatment failure due to poor quality of life may not be able to go to work. They may have to be hospitalised for management of HIV related opportunistic infections and other complications. This could cause economic hardship. It might take some time for them to reach a level of stability on treatment. Due to the uncertainty involved during this period the quality life may be adversely impacted.

In this study more than 50% PLWHA had a history of Tuberculosis. Studies in the past have identified active Tuberculosis as an important determinant of poor quality of life in PLWHA^35^. The risk of Tuberculosis activation increases due to poor immunity associated with HIV treatment failure. Additionally, patients on cART and anti-Tuberculosis treatment are at a risk of adverse drug reactions and interactions. This can lead to a decline in the quality of life.

Limitations: This study has several limitations. First, its observational design limits causal inference. Additionally, the study was not specifically powered to examine all HIV-related and social determinants of HRQoL, including those that may explain the observed gender differences. While the EQ-5D-5 L instrument offers greater domain-specific sensitivity than the EQ-VAS, it may still be influenced by factors not captured in our analysis. Second, all participants were receiving Zidovudine- and Efavirenz-based cART regimens, as per national guidelines in effect during the study period. At that time, routine viral load–based switching was not yet standard practice. As a result, the applicability of these findings to current treatment protocols may be limited. Third, the study overlapped with the COVID-19 pandemic, which may have influenced patient follow-up, psychosocial well-being, and HRQoL. These potential confounders were not independently assessed.

## Conclusions

This study, despite its limitations, has some conclusive findings. EQ-5D-5 L index values decreases significantly in PLWHA with treatment failure. Female gender and a declining recent CD4-T cell count are among the key predictors of low EQ-5D-5 L index value in PLWHA on treatment. Although viral load based monitoring is a standard now, early switching to a new regimen is the key to the success of changed treatment. Following treatment guidelines and HIV viral load-based strategies for early switch to a potent new regimen in PLWHA with treatment failure, adverse impact on quality of life may be reduced. Future studies should evaluate factors that influence HRQoL differences between males and females.

## Supplementary Information

Below is the link to the electronic supplementary material.

Supplementary Material 1

## Data Availability

The datasets generated and/or analysed during the current study involve sensitive clinical information from people living with HIV (PLHIV). Due to privacy and ethical considerations, these data are not publicly available. However, de-identified datasets may be made available from the corresponding author upon reasonable request and with appropriate institutional approvals.
