# Supplementary material for: Clinical and quality of life consequences of regimen switching delays in HIV management: a stratified cohort analysis
Source: Sci Rep. 2025 Nov 29;15:45727. doi: 10.1038/s41598-025-28209-w (PMC12753822; doi:10.1038/s41598-025-28209-w)
Supplement: Supplementary file 1 — Supplementary Material 1 [file 41598_2025_28209_MOESM1_ESM.docx]

**Supplementary Material 1**

Detailed Scoring Methodology for EQ-5D-5L Questionnaire and EQ-VAS

**B. Scoring of EQ-5D-5L Questionnaire and EQ-VAS**

The EQ-5D-5L questionnaire was employed to assess the health-related quality of life (HRQoL) of participants[14], utilizing a comprehensive five-dimensional descriptive system. The five domains assessed by the EQ-5D-5L include:

Mobility

Self-care

Usual activities

Pain/discomfort

Anxiety/depression

Each domain was evaluated on a five-point Likert scale, representing varying levels of perceived health problems:

Level 1: No problems

Level 2: Slight problems

Level 3: Moderate problems

Level 4: Severe problems

Level 5: Extreme problems

Participants selected the level that best represented their perceived health status for each dimension. The combination of one level from each of the five domains created a 5-digit health state code (e.g., 11111 for perfect health). For example, the code 12345 would indicate no mobility issues, slight self-care problems, moderate issues with usual activities, severe pain/discomfort, and extreme anxiety/depression.

Only one response per domain was considered valid. Multiple responses or missing entries were coded as ‘9’ to denote incomplete or invalid responses. Ambiguous answers were similarly treated as missing data.

The EQ Visual Analogue Scale (EQ-VAS) was used in conjunction to further quantify self-reported health status[15]. Participants rated their health on a scale from 0 (worst imaginable health) to 100 (best imaginable health), providing a complementary quantitative measure to the EQ-5D-5L.

Notably, the numeric values in the EQ-5D-5L (e.g., 21111 vs. 13111) are not arithmetically comparable, as each domain describes distinct qualitative states.

To calculate an overall EQ-5D index, responses were weighted based on pre-established valuations. The index was computed by subtracting the weighted values from 1 (representing perfect health). This allowed for standardized comparison across participants’ HRQoL scores.

In this study, the crosswalk link function by Van Hout et al. (2012) [16] was applied to derive EQ-5D-5L index values from participants’ 5-digit profiles. This method, based on the EuroQol Research Foundation’s guidance, produced values ranging from 0 to 1 (e.g., 0.88 for high HRQoL, 0.43 for lower HRQoL).

To enhance accessibility, the EQ-5D-5L and EQ-VAS instruments were provided in English, Kannada, and Malayalam, allowing participants to self-complete them in their preferred language.

**Operational Definitions**:

1. **Patient groups:** The enrolled patients were divided into three groups based on the most recent two consecutive viral load reports and the cART at the time of enrolment.
2. **Stable First-line cART Group:** On stable first line cART with HIV RNA viral load <1000 copies/ml
3. **First-line Failure & Early Second-line Group:** On first line cART with HIV RNA viral load >1000 copies/ml or on second line cART for less than a year
4. **Stable Second-line Group:** On stable second line cART with HIV RNA viral load <1000 copies/ml
5. **Treatment (cART) failure:** Two consecutive viral load values more than 1000 copies/ml after being on regular cART for more than six months with adherence more than 95%.
6. **Stable cART:** Fulfilling all the criteria

* Receiving cART for at least one year

* No adverse drug reactions requiring regular monitoring

* No current illnesses or pregnancy

* Good understanding of lifelong adherence

* Evidence of treatment success: Two consecutive undetectable viral load measures (or, in the absence of viral load monitoring, rising CD4-T cell counts or CD4-T cell counts above 200 cells/mm^3^ and objective adherence measure).

| Variable | Total  n=321(100%)  n (%) | cART Groups | | | X^2^ &  *p* Value |
| --- | --- | --- | --- | --- | --- |
|  |  | First line stable  n = 203 | First line failure & Early second line  n = 31 | Second line stable  n = 87 |  |
| HIV Duration:  Mean duration in Years  cART Duration:  Mean duration in Years | 9.4 (±4.7)  8.2 (±4.2) | 9.2 (±4.7)  8.1(±4.3) | 9.1(±5.9)  7.2(±4.7) | 9.7(±4.4)  8.6(±3.9) | *p* = .677  *p* = .275 |
| CD4 @ HIV diagnosis ≤350  CD4 Cell count: (Median)  @ HIV Diagnosis  Last 18 months  Last 12 months  Last 6 months | 230 (71.7%)  219(IQR 119-373)  483(IQR 300-655)  505(IQR 339-657)  496(IQR 349-665) | --  214(IQR111-359)  576(IQR405-736)  559(IQR434-709)  560(IQR426-741) | --  154(IQR87-327)  300(IQR131-436)  284(IQR151-408)  231(IQR134-482) | --  254(IQR144-410)  380(IQR212-554)  401(IQR240-549)  443(IQR287-598) | --  *p* = .133  *p* = <.001*  *p* = <.001*  *p* = <.001* |
| Min Viral load (c/ml)  > 6months:  Viral load <40:  Viral load 40-1000:  Viral load >1000:  Recent Viral load (c/ml)  < 6months:  Viral load <40:  Viral load 40-1000:  Viral load >1000 | 252(78.5%)  40 (12.5%)  29(9%)  281(87.5%)  12(3.7%)  28(8.7%) | 185(91.1%)  18(8.9%)  0(0%)  198(97.5%)  5(2.5%)  0(0%) | 3(9.7%)  1(3.2%)  27(87.1%)  3(9.7%)  0(0%)  28(90.3%) | 64(73.6%)  21(24.1%)  2(2.3%)  80(92%)  7(8%)  0(0%) | L.R/X^2^ =164.01  *P* = <.001**  L.R/X^2^  =174.94  *P* = <.001** |
| cART Regimen:  Initial:  AZT/NNRTI  TDF/NNRTI  TDF/PI  Other  Current:  AZT/NNRTI  TDF/NNRTI  AZT/PI  TDF/PI  Others | 210(64%)  104(32%)  4(1.2%)  3(0.8%)  124(38.6%)  84(26.2%)  70(21.8%)  31(9.7%)  12(3.7%) | 140(69%)  60(29.6%)  2(1%)  1(0.5%)  119(58.6%)  74(36.5%)  3(1.5%)  0(0%)  7(3.4%) | 16(51%.5%)  11(35.5%)  2(6.5%)  2(6.5%)  4(12.9%)  8(25.8%)  12(38.7%)  2(6.5%)  5(16.1%) | 54(62.1%)  33(37.9%)  0(0%)  0(0%)  1(1.1%)  2(2.3%)  55(63.3%)  29(33.3%)  0(0%) | L.R/X^2^  =14.99  *p* = .02**  L.R/X^2^  =321.0  *P*= <.001** |
| History of TB/OI:  Yes  No | 161(50.2%)  160(49.8%) | 77(37.9%)  126(62.1%) | 21(67.7%)  10(32.3%) | 63(72.4%)  24(27.6%) | X^2^=33.21  *P*=<.001** |

**Results**

**Table 1b: Treatment History and Immunovirological Profile of Study Participants**

*Kruskal-Wallis H test, ** Chi-square test

**Note: All regimens included a dual NRTI backbone as per national guidelines.**

**Initial Regimen Definitions:**

- AZT/NNRTI = Zidovudine (AZT) + Lamivudine (3TC) + Nevirapine or Efavirenz

- TDF/NNRTI = Tenofovir (TDF) + Lamivudine (3TC) + Efavirenz

- TDF/PI = TDF + 3TC + Atazanavir/ritonavir or Lopinavir/ritonavir

**Current Regimen Definitions:**

- AZT/NNRTI = AZT + 3TC + Nevirapine or Efavirenz

- TDF/NNRTI = TDF + 3TC + Efavirenz

- AZT/PI = AZT + 3TC + Dolutegravir (DTG) or a PI

- TDF/PI = TDF + 3TC + DTG
